# Burns: Classification, Pathophysiology, and Treatment: A Review

**DOI:** 10.3390/ijms24043749

**Published:** 2023-02-13

**Authors:** Wojciech Żwierełło, Krzysztof Piorun, Marta Skórka-Majewicz, Agnieszka Maruszewska, Jacek Antoniewski, Izabela Gutowska

**Affiliations:** 1 Department of Medical Chemistry, Pomeranian Medical University, 70-204 Szczecin, Poland; 2 West Pomeranian Center for Treating Severe Burns and Plastic Surgery, 72-300 Gryfice, Poland; 3 Department of Physiology and Biochemistry, Institute of Biology, University of Szczecin, 71-412 Szczecin, Poland

**Keywords:** burns, pathophysiology, treatment

## Abstract

Burns and their treatment are a significant medical problem. The loss of the physical barrier function of the skin opens the door to microbial invasion and can lead to infection. The repair process of the damage caused by the burn is impaired due to the enhanced loss of fluids and minerals through the burn wound, the onset of hypermetabolism with the concomitant disruption of nutrient supply, and derangements in the endocrine system. In addition, the initiated inflammatory and free radical processes drive the progression of oxidative stress, the inhibition of which largely depends on an adequate supply of antioxidants and minerals. Clinical experience and research provide more and more data to make the treatment of patients with thermal injury increasingly effective. The publication discusses disorders occurring in patients after thermal injury and the methods used at various stages of treatment.

## 1. Introduction

Skin is the human body’s largest organ, covering a surface area of about 2 sqm in an average adult. It consists of the epidermis and the dermis, deep within which are important skin appendage structures (including hair follicles, sweat glands and sebaceous glands). These deep structures are a source of proliferating epithelial cells (keratinocytes), which migrate into the clot and wound bed, playing an important role in the wound healing process. The loss of the physical barrier function of the skin opens the door to invasion by harmful microorganisms, which can lead to infection, and ultimately even to the development of sepsis. The repair process of burn injury, which begins as early as several hours after the traumatic event, may also be impaired by large fluid losses via the wound [1]. Any burn, even relatively minor, can have functional and aesthetic implications lasting throughout the patient’s lifetime.

Burns and their treatment have been regarded as an important medical problem since antiquity. The first formulations for concoctions to be used in burn care can be found already in prehistoric paintings, Egyptian papyri, and ancient Chinese art. The historical writings of Hippocrates, Celsus, and Galen describe increasingly elaborate methods for making ointments, dressings, and treatment regimens for different types of burns. In the mid-16th century, Ambrose Paré was one of the first to describe early burn wound excision. At the beginning of the 17th century, Guilhelmus Fabricius Hildanus ventured to discuss the pathophysiology of burns, making a unique contribution to the treatment of scar contractures, among other things. In 1797, Edward Kentish described the use of pressure dressings to alleviate the effects of burns and blistering, while in 1839, Dupuytren reviewed more than 50 cases of burns and presented a classification with six degrees of burn depth. His classification is still in use in many parts of the world. In the twentieth century, major developments in our knowledge of burn care occurred, particularly with regard to the problems of fluid loss and resuscitation, the hypermetabolic response to burns, infection control and the development of topical antimicrobials, early excision of burned tissue and wound closure with autologous or allogeneic skin grafts, keratinocyte culture, and, last but not least, the use of artificial skin substitutes. Efforts aimed at advancing our understanding of the problem of burns are gradually improving survival rates and the quality of life of burn patients. This does not change the fact that many aspects of the pathophysiology of this type of injury need further research, which will make it possible to develop a better, standardised, and generally accepted effective burn resuscitation regimen [2,3].

The use of appropriate treatment strategies in the shortest possible time from the occurrence of thermal injury can not only save the patient’s life, but also shorten their hospital stay and recovery time. Therefore, the aim of the study was to comprehensively discuss the disorders occurring in patients at different times after the occurrence of burns and the appropriate treatment methods.

A literature analysis was carried out on the PubMed database. The following keywords were used to search for available articles: “Burns”, “Burn”, “Burns pathophysiology”, “Burns treatment”, “Burn injury”, “Thermal injury”, “Burns treatment”. The time range of the searched articles was not established. We tried to use the latest reports on the pathophysiology and treatment of burns, but when discussing changes in the patient’s body after thermal injury, we did not want to eliminate older reports describing significant metabolic changes. Filters related to the type of articles (clinical trials, review, systematic review, book) were used. Repetitions were rejected from the found articles. The suitability for the inclusion of each study into the publication was thoroughly assessed. Eventually, 83 articles were included in the review.

## 2. Burn Injury

A burn injury results from skin contact with a heat source [4]. The factors that can cause burn injuries include high temperature, electricity, friction, radiation and chemicals [5]. Burn injuries vary, and an increase in the body surface area affected by the burn injury affects wound morbidity and patient mortality [6]. Other important factors directly impacting on the severity of injury include the location of the burn, temperature and time of exposure to the heat source, with a synergistic effect between them [7].

### 2.1. Classification of Burns

Burn injuries can be classified according to a number of factors, including their depth, aetiology and percentage of body surface area affected. The combination of the above classifications determines the degree of burn injury. Burns can be classified as “partial-thickness” and “full-thickness”. If the damage is limited to the epidermis and the outer part of the dermis (a superficial partial-thickness burn), with most of the appendage structures remaining intact, recovery will be rapid (10–14 days) and the risk of scarring low. If, on the other hand, the burn extends into the deeper layers of the dermis, with greater appendage damage, the epithelium will take longer to regenerate (3–6 weeks) and there will be a high probability of hypertrophic scarring. Full-thickness burns involve the destruction of all layers of the skin and usually require surgical intervention to ensure proper wound healing [1,8].

### 2.2. Aetiology

The origin of burn injuries can be thermal, electrical, chemical, radiation contact, etc.

#### 2.2.1. Thermal Injuries

**Figure 1:**
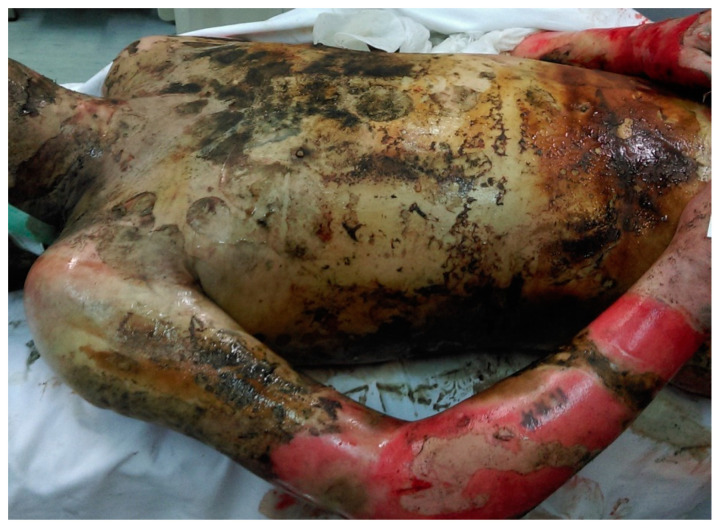
A full-thickness 3rd degree thermal burn.

Thermal injuries account for about 90% of all burns, and the depth of injury depends on the temperature and duration of contact (Figure 1). They can be divided into:

- Injuries caused by hot liquids (scalds)—the most common type of burn injury, accounting for nearly 70% of burns in children, but also common in the elderly. Scalds usually cause partial-thickness burns that heal after a standard treatment regimen;
- Dry heat injuries—usually caused by direct contact with a flame or radiant heat. Common in adults and often associated with complications due to smoke inhalation. They are usually deep (partial or full thickness) and generally require surgical intervention;
- Contact injuries—result from direct contact with a hot object. Prolonged contact with a moderately hot object (e.g., a radiator) can also cause a thermal injury, which is commonly associated with loss of consciousness (e.g., in the elderly, patients with epilepsy, drug addicts and alcoholics). Contact burns are usually deep and require surgery [9].

#### 2.2.2. Electrical Injuries

Electrical injuries account for less than 5% of all burns. They are most common in children and male manual workers. The severity of injury is determined by the voltage and amperage, the type of current, duration of contact, and the pathway of the current through the body. Most tissues are good conductors, especially nerves and vessels. Skin and bones are poor conductors, although skin conductivity varies depending on its moisture content and temperature. The heat is generated by electricity around the tissues that are poor conductors, damaging the local surrounding tissues. Clinically, one often observes the so-called entrance and exit points, where the electrical current has passed through the body. An electrical voltage of <1000 V, typically found indoors, causes small, deep burns at the entrance and exit points. Alternating current can also interfere with heart function and lead to arrhythmias. High-voltage injuries (>1000 V) lead to extensive tissue damage, often with loss of limbs, asystole, cardiac arrhythmia, rhabdomyolysis (muscle breakdown) and renal failure. Fluid resuscitation is complex due to the invisible nature of the injuries. This type of injury is associated with a high mortality rate, and approximately 15% of victims have additional injuries from falls [10].

Burns can also be caused by the mere arc flash of a discharge between high-voltage sources. While current does not pass through the body, the heat from the arc can burn exposed parts of the body (hands and face). The resulting burns are usually partial-thickness, unless the arc causes clothing to ignite, leading to deeper injuries [11].

#### 2.2.3. Chemical Injuries

Chemical injuries account for approx. 3% of burns. Incidents of this type mainly occur in domestic and industrial settings. This type of injury involves denaturation of proteins and the extent of injury depends on the concentration, amount, duration of contact, and mechanism of action of the given chemical, i.e., reduction and oxidation, corrosion, protoplasmic poison, vesication, and desiccation. While the clinical picture is similar for all groups of chemicals, the exact mechanisms of tissue damage may vary, hence chemicals have traditionally been classified into acids or alkalis [12].

Acid burns cause damage resulting in protein denaturation and necrosis, which is usually localised and short-lived. Alkaline burns, on the other hand, cause progressive liquefaction necrosis, with deeper tissue penetration and a prolonged effect. Cement causes alkaline burns, and when mixed with sweat it can induce an additional exothermic reaction. In addition, cement powder is highly hygroscopic and causes severe desiccation of the affected surface. Washing with copious amounts of water dilutes the chemical and helps reduce tissue damage [12]. Burns are most likely to be caused by acids (sulphuric, nitric, hydrofluoric, hydrochloric, acetic, formic, phosphoric, phenolic and chloroacetic acids), alkalis (sodium hydroxide, potassium hydroxide, calcium hydroxide and lithium hydroxide, sodium and calcium hypochlorite, ammonia, phosphate, silicate, sodium carbonate), oxidisers (bleaches such as chlorites used in the household, peroxides, chromates), or other chemicals (white phosphorus, hair colouring agents, mustard gas).

#### 2.2.4. Radiation

Generally, harmful radiation is caused by alpha (α), beta (β) and gamma (γ) rays. Alpha particles are positively charged helium ions. They are heavy, can only travel a few centimetres in the air, and cannot penetrate the keratin layer of the skin. However, these are high energy particles with high Sv (sievert) value and can cause extensive tissue damage upon ingestion or inhalation. Beta particles are negatively charged electron beams that can travel several metres in the air and cause superficial sunburn-like injuries because of their limited ability to penetrate deep into tissue (1 cm) [13].

Gamma rays from X-rays and the natural decay of radioisotopes, such as ^60^Co (cobalt) and ^192^Ir (iryd), can travel several metres in the air and penetrate deep into tissues. Consequently, gamma rays can cause very deep damage involving vital structures such as the bone marrow and lungs. In addition to deep gamma burns on the skin, patients experience systemic symptoms described as Acute Radiation Syndrome (ARS) [13].

## 3. Pathophysiology of Burn Injuries

### 3.1. Local Effects of Burn Injuries

Burn injuries cause coagulative necrosis of various layers of skin and underlying tissues. Because of its main function as a physiological barrier protecting underlying tissues, the skin usually limits the spread of damage to deeper layers, but the extent of damage is determined by the temperature, the energy transmitted by the causative agent, and the duration of exposure [14]. In principle, the site of a cutaneous burn injury can be divided into three zones:

- Zone of coagulation—represents the area of necrosis with irreversible tissue damage incurred at the time of injury;
- Zone of stasis—surrounds the coagulation zone and is moderately damaged with vascular transudate, elevated vasoconstricting factors, as well as local inflammatory reactions, resulting in impaired tissue perfusion. Depending on the wound environment, the zone may recover or progress to necrosis;
- Zone of hyperaemia, with dilated vessels caused by inflammation. It is characterised by increased blood flow to healthy tissues without much risk of necrosis, unless there is severe sepsis or prolonged hypoperfusion [14].

### 3.2. Systemic Effects of Burn Injuries

Burns involving more than 30% of total body surface area (TBSA) result in considerable hypovolemia coupled with the formation and release of inflammatory mediators, leading to a subsequent systemic effect, namely a characteristic cardiovascular dysfunction known as burn shock. It is a complex process of circulatory and microcirculatory impairment, generating oedema in both burned and unaffected tissues. Even with prompt intervention and adequate fluid support, this pathophysiological state remains completely irreversible [15]. Plasma extravasation is another feature of burn injury, resulting in increased systemic vascular resistance (SVR) and reduced peripheral blood flow. This results in hemodynamic changes, which include a reduction in cardiac output due to the diminished plasma volume, as well as a decrease in urinary excretion [16,17].

Burn shock involves a state of inadequate tissue perfusion with resultant inadequate oxygen and nutrient delivery, as well as failure to remove metabolic waste from the tissues [15]. Despite proper fluid resuscitation and adequate preload, pulmonary and systemic vascular resistance increases and myocardial depression occurs. This in turn stimulates further exacerbation of the inflammatory response and contributes to the risk of multiple organ failure [15]. Importantly, elevated haemoglobin and haematocrit levels are also observed in burn injuries [16,17].

Another response of the body to a burn is oedema formation. Enema develops when the amount of fluid filtered out of microvessels is greater than the amount of fluid entering them [15]. The process of oedema formation is biphasic. The primary phase, initiated in the first hour after the burn injury, is caused by a rapid increase in the water content of the damaged tissues. The second phase, occurring 12–24 h after the burn injury, involves a slower, gradual increase in fluid flow in both the burned and intact skin and soft tissues [15,16].

In the development of post-burn oedema, an important role is played by the rate of increase in tissue water content, which is clearly influenced by the type and amount of fluid resuscitation administered to the patient. The tissue water content reaches double the original volume within the first hour, with 90% of the increase observed in the first few minutes. The use of fluid resuscitation contributes to further extravasation, influenced by increased blood flow and increased capillary pressure under the influence of the delivered fluids. On the other hand, oedema tends to be self-limiting when fluids are not administered [18,19].

In burn injuries exceeding 30% TBSA, thermal insults result in a decrease in the cellular transmembrane potentials in skeletal muscles not only at the site of injury, but also distant to the site of injury [20]. It has been shown that cell membranes in damaged and intact skeletal muscles demonstrate partial depolarisation of membrane potential from −90 mV to −80mV and −70 mV. As soon as there is a decline in membrane potential, the water and sodium content within cells increases. These alterations are also seen in haemorrhagic shock. Similar changes have been reported in cardiac, hepatic and endothelial cells [20]. Some scholars have linked membrane depolarisation to a decrease in adenosine triphosphate (ATP) and reduced ATPase activity in the respiratory chain. Others suggest that the mechanism depends on increased membrane permeability to sodium ions, associated with increased Na/K pump activity [21]. Research aimed at identifying the factors responsible for the cellular oedema seen in burn shock postulated the existence of unidentified and complex shock factor(s) [21]. This hypothesis was supported by Button et al., who demonstrated that burn-associated tissue oedema cannot be attributed solely to hypovolemia, and that burn shock should not be regarded as another form of haemorrhage [22].

The enormous energy demand, measured by resting energy expenditure, is a typical finding in burn patients, with the increase in metabolism (hypermetabolism) dependent on the size of burn. In patients with a TBSA of less than 10%, resting energy expenditure remains at physiological levels, but for TBSA in excess of 40%, this rate is twice as high during acute admission. Having reached the maximum value, the resting metabolic rate in severely burned patients gradually declines, amounting to 150%, 140%, 120% and 110% of baseline at the time of burn wound healing, 6, 9 and 12 months after thermal injury, respectively [23].

Underlying the hypermetabolic response following thermal injury are mechanisms of metabolic, hormonal and inflammatory dysregulation. This is a highly complex phenomenon, triggered by persistent increases in the secretion of catecholamine, cortisol, glucagon, and dopamine, and elevated concentrations of interleukin 1 (IL-1), interleukin 6 (IL-6), tissue necrosis factor (TNF), platelet-activating factor (PAF), complement cascades, as well as increased synthesis of reactive oxygen species (ROS) [24]. These metabolic regulations were found to occur in two phases: early (ebb) and late (flow). The “ebb” phase begins immediately after thermal injury and lasts approximately three days. It is characterised by hypodynamic circulation, reduced oxygen consumption and hyperglycaemia. These variables then begin to increase progressively until reaching the “flow” phase, which usually lasts up to a year since the burn injury [25].

The body’s hypermetabolic response has detrimental effects at the cellular and systemic level [26]. At the systemic level, the structure and function of major organs (heart, liver, skeletal muscle, skin), the immune system and the transmembrane transport system are compromised. Wound healing is impaired, which increases the risk of infection, hampers rehabilitation and delays the reintegration of patients back into society [26,27]. At the cellular level, hypermetabolic response increases thermogenesis [28] by uncoupling mitochondrial respiration from phosphorylation of ADP to ATP, resulting in heat generation [29]. Simultaneously, increased energy demand enhances oxygen consumption [28]. The adipose tissue of burn patients was reported to contain elevated levels of uncoupling protein 1 (UCP1), an important mediator of thermogenesis [30,31].

The endocrine disruption that occurs after a burn alters metabolic pathways. Catecholamines drive hypermetabolism, while an increase in the secretions of cortisol, adrenaline and glucagon (which are catabolic hormones), together with an increase in pro-inflammatory cytokines, inhibits protein and fat synthesis [26,32]. The observed negative nitrogen balance in burn patients [25] suggests that skeletal muscles are used as the main energy source [33]. Accelerated protein degradation leads to a significant loss of lean body mass (LBM) and muscle atrophy, resulting in reduced strength and compromised rehabilitation outcomes [26,34]. Depending on the magnitude of LBM loss, certain dysfunctions occur. While alterations in the immune system, increased rates of infection and delayed wound healing are correlated with a 20% loss of LBM, patients with a 30% loss of LBM present inhibited cough reflexes, prolonged requirements for mechanical ventilation, as well as an increased risk of pneumonia and pressure sores. With LBM loss reaching 40%, mortality among burn patients goes up to 50–100% [35].

Research has shown that impaired glucose metabolism can still be seen up to three years after thermal injury [24]. In severe burns, hypermetabolism and oxygen deprivation in the cells lead to anaerobic glycolysis, where glucose is converted to lactic acid [36]. In addition, patients with severe burns exhibit increased glucose production through activation of the gluconeogenesis pathway, with alanine as the main substrate (next to lactic acid). In this situation, amino acids become the main “fuel”, resulting in a deficit of amino acids for building proteins, as well as an increase in nitrogen excretion, mainly in the form of urea [26].

An increase in gluconeogenesis activity associated with an increase in gluconeogenic substrates, which include glycerol (derived from the breakdown of triacylglycerols), alanine (derived from the breakdown of proteins) and lactate (a product of anaerobic glycolysis), leads to hyperglycaemia in patients with severe burns. Research has shown that serum glucose levels are persistently elevated in these patients, reaching of up to 180 mg/dL. This condition is further compounded by an attenuation of the suppressive effect of insulin on hepatic glucose release and enhanced hepatic glycogenolysis [37]. Interestingly, determination of insulin levels in serum samples (showing a twofold increase) points to the development of insulin resistance in these patients [37].

Thermal injury also triggers changes in the circulatory system. Cardiac function is subject to several modifications starting already at the time of injury. Before detecting any reduction in plasma volume, receptors on thermally damaged skin trigger a neurogenic response, initiating a rapid decrease in cardiac output. This is associated with an initial reduction, followed by a significant increase in the cardiac index starting on the third day post-burn [25]. Other parameters, such as long-term increase in cardiac work, increased myocardial oxygen consumption, and heart rate acceleration, remain elevated during the recovery period [38]. Severe cardiac stress is accompanied by a persistent myocardial depression that can be attributed to hypovolemia, high SVR, low venous return and the effects of myocardial depressant substances. Fluid resuscitation usually fails to restore normal cardiac output.

Urinary dysfunction is a consequence of alterations in cardiovascular function and endocrine dysregulation (changes in angiotensin, vasopressin and aldosterone secretion). The development of hypovolemia, as well the diminished cardiac output following thermal injury bring down the glomerular filtration rate (GFR) as a result of reduced renal blood flow. These alterations usually manifest themselves in the form of oliguria, and if not managed promptly and appropriately it can lead to acute tubular necrosis (ATN), renal failure and even death [39].

Following thermal injury, it is critically important to provide for an adequate nutrient supply to meet the increased energy expenditure that occurs due to the hypermetabolic response. However, the digestive process is impaired in proportion to the magnitude of the burns. Due to the apoptosis of enterocytes (the cells making up the intestinal epithelium) and mucosal atrophy, absorptive capacity is reduced, particularly the uptake of glucose, amino acids and fatty acids, and the situation is further compounded by changes in the secretion and activity of digestive enzymes, including pancreatic lipase, involved in lipid digestion. In addition, with increased intestinal permeability, undesirable compounds can pass from the intestinal tract into the bloodstream [40].

Thermal trauma also disrupts liver function. Research has shown that thermal injury alters hepatic expression and serum concentrations of acute phase proteins. Serum complement C3 and α2-macroglobulin concentrations in burn patients initially fall, and then gradually rise. The redirection of substrates to synthesise these proteins, the increased use of muscle proteins for energy production due to the hypermetabolic response and the impaired absorption of nutrients (including amino acids) in burn patients are the likely factors suppressing the synthesis of constitutive hepatic proteins [25]. Lower production of the protein components for VLDL lipoproteins (transporters for triacylglycerols and fatty acids) reduces their release from the liver, which can lead to the fatty infiltration of this organ. This in turn increases the risk of sepsis. In addition, the use of TG as an energy substrate in extrahepatic tissues is reduced [25].

Endocrine response is one of the systemic reactions observed in severely burned patients and is characterised by significant functional alterations in the hypothalamic-pituitary axis. During the early post-burn phase, there is a marked upsurge in so-called stress hormones, which include catecholamine, glucagon, and cortisol [41]. They are thought to be the initiators of the body’s hypermetabolic, catabolic, and proteolytic response to the burn injury, with significant effects on cardiovascular function and resulting changes in water-electrolyte balance. Substantial changes are also observed in the production of thyroid hormones: TSH (thyroid-stimulating hormone), T3 (triiodothyronine), T4 (thyroxine) and parathyroid hormone (PTH), as well as testosterone and osteocalcin, whose serum concentrations were found to decrease in patients with thermal injuries. The initial stress-related hormonal response is followed by alterations at several points in the hypothalamic-pituitary-organ axes. Notably, severe burns cause specific modifications in the GH (growth hormone)—IGF-1 (insulin-like growth factor 1) axis. Significantly, the concentrations of IGF-1 and insulin-like growth factor binding protein-3 (IGFBP-3) were found to be more profoundly affected than GH [42,43].

Scholars have also demonstrated the effects of burns on the male reproductive system. Thermal injuries tend to affect the histology of the seminiferous epithelium with germ cell atrophy. A number of factors play a role in the aetiology of germ cell apoptosis and altered spermatogenesis: increased temperature in the scrotum, decreased hormone synthesis, systemic trauma, and oxidative stress subsequent to inadequate perfusion. Decreased blood testosterone levels are sometimes attributed to the presence of testicular toxicants. The harmful effects of these substances can be reversed by administering antioxidants, including ascorbic acid, which simultaneously reduces the body’s resuscitative fluid needs [43,44].

In the immunological context, thermal insults have a significant comprehensive impact on the immune system, particularly the cellular immune response. Immune deficiency in burn patients is thought to be caused by impaired expression of bone marrow granulocyte colony-stimulating factor (*G-CSF*) receptors. While the loss of skin and the mechanical barrier facilitates infection in patients with burn injuries, it has long been known that impaired immune mechanisms are key factors in bacterial, viral and fungal infections following burn injury [45].

## 4. Treatment of Patients after Thermal Injury

While none of the established therapeutic approaches to date have been able to completely reverse the complex reactions induced by burns, there is a number of pharmacological and non-pharmacological strategies which are effective in modulating burn-associated metabolism.

### 4.1. Cooling of Burned Areas

Research has shown that in the event of a burn, immediate removal of the cause and cooling of the injured area is beneficial to the burn victim. Reducing the elevated temperature of the burned tissue improves the physiological response. Importantly, it also provides palliative relief. The cooling agent should be applied as promptly as possible, but it must be at the right temperature. Extreme cold (e.g., ice) can cause further damage by reducing blood flow to the injured area (cold-induced vasoconstriction). Cooling of a large area of skin over a long period of time is likely to induce hypothermia. There is also a risk of frostbite on cooled surfaces. According to the available literature, the optimal temperature for cooling a burn injury is 10–20 °C [46].

### 4.2. Fluid Resuscitation

In the event of a severe burn, the first and most important therapeutic intervention is adequate resuscitation [47]. After a burn injury, fluid rapidly accumulates in damaged tissues and, to a lesser extent, in healthy tissues. Without resuscitation, burns greater than 15–20% TSBA can lead to hypovolemic shock, organ dysfunction and ultimately death of the victim. The 24-h fluid requirements of a burn victim are estimated using the Parkland formula for fluid resuscitation, which remains the most widely used protocol worldwide to date. Since its introduction by Baxter and Shires in 1968, it has become the gold standard for initial fluid resuscitation in burns [48]. The formula, based on the patient’s weight and the percentage of body surface area burned, is used in combination with regular measurements of physiological parameters and resuscitation endpoints, particularly the urine output. The Parkland formula estimates the total fluid requirement over 24 h at 4 mL/kg/% TBSA, with half of this volume to be administered in the first 8 h. In the past decade, concerns about the accuracy of this formula have led clinicians to re-evaluate the fluid resuscitation process, particularly in elderly patients. The concept of excessive resuscitation was addressed by in Pruitt in his 2000 report [49].

The phenomenon of excessive fluid loading usually results from a combination of several factors, i.e., inaccuracies in calculating fluid requirements, unnecessary fluid infusions, increased use of sedation and analgesic infusions, and excessive administration of crystalloid solutions [47]. In order to improve the accuracy of fluid resuscitation, attempts are being made to introduce adjunctive measures in the form of modern minimally invasive procedures, such as the insertion of a pulmonary artery catheter or translung thermodilution, allowing for continuous monitoring of venous oxygen saturation, intrathoracic blood volume, total blood volume index and extravascular lung water index, but irrespective of the above urine output remains the main indicator of adequate fluid resuscitation. Isotonic crystalloid resuscitation fluids (lactate or acetate Ringer’s solution) are recommended for fluid resuscitation. The simultaneous use of colloid and hypertonic lactated saline (HLS) is recommended as an option for fluid resuscitation [50].

### 4.3. Ventilation

Airway management and ventilator support are often required in cases of severe burns, particularly in thermal lung injuries. Ventilation strategies for respiratory failure in critically ill patients, including those with severe burns, are still being developed. The introduction of a lung-protective ventilation strategy has reduced the incidence of ventilator-associated lung damage. Overall technological advances in the field of ventilation have shown measurable improvements in outcomes for patients with severe burns and inhalation injuries [51].

### 4.4. Surgical Treatment

Early excision and closure of the burn wound is sometimes described as the greatest advance in the treatment of patients with severe thermal injuries. In fact, this strategy is crucial for reducing the incidence of complications associated with severe burns [52]. The metabolic rate in patients undergoing total excision and wound coverage with an autograft and/or deceased donor skin within the first 72 h following severe thermal injury (50% TBSA) is 40% lower than the metabolic rate in patients with similar burn severity who did not undergo excision within a week. Immediate excision also offers additional advantages, which include reduced protein loss, lower risk of infection and sepsis, and less pain compared to patients with delayed reconstruction [52].

**Figure 2:**
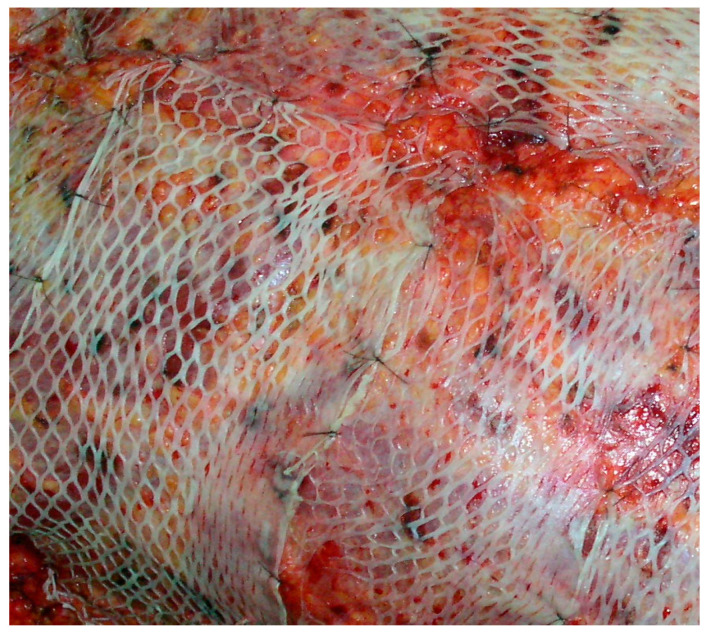
Intermediate-thickness skin grafts on a burn wound.

Reconstructive burn surgery has greatly improved the quality of life for burn patients by restoring function and appearance to the affected areas. This type of surgery may involve skin grafts (Figure 2), tissue expansion, and other techniques to repair damaged tissue and minimise scarring [53,54].

Eligibility for reconstructive burn surgery depends on several factors, including the extent and location of the burn, the patient’s overall health, and the presence of other medical conditions. In general, patients with burns that affect functional or cosmetically significant areas of the body, such as the face, hands, and feet, may be good candidates for reconstructive surgery. The timing of the surgery is also important and is usually carried out after the burn wounds have healed [54].

Assessment of burn depth poses a major challenge even to experienced surgeons, as there are no precise methods to do so that can be used at an early stage (up to several days after the injury). Physicians can take guidance from a few important clues, such as the mechanism of the burn injury, redness or sensory preservation in the tissues, but such an assessment is subject to considerable error. That is why Laser Doppler Imaging (LDI), an accurate diagnostic tool with high sensitivity and specificity, has proven to be an important adjunct to clinical assessment. It is used to measure the degree of disruption of dermal microvascular blood flow and makes it possible to assess total depth with a high degree of accuracy. The use of LDI has resulted in shorter hospital stays, lower rates of surgical interventions, shorter decision-making times for grafting procedures, and overall cost efficiency [55]. Another prospective assessment method may be active dynamic thermography, where the temperature of the burn wound is measured as an indicator of its depth [56].

In recent decades, many innovative techniques have been introduced to improve the surgical treatment of burn wounds. The use of various skin substitutes has been particularly important in the evolution of burn surgery, providing recovery options in injuries previously considered impossible to reconstruct. Skin substitutes are a diverse group of wound-covering materials. They are intended to help close the wound when autologous skin grafts are either unavailable, e.g., in extensive burns, or undesirable, e.g., in full-thickness burns with significant loss of dermis [57]. In addition to rapid wound closure, they help increase the dermal component of the treated wound, reduce or eliminate the factors that inhibit healing, reduce the inflammatory response, and, consequently, the risk of scar formation [58].

Skin substitutes have been categorised into temporary impervious dressing materials (Class I), single-layer durable epidermal or dermal substitutes (Class II), and composite skin substitutes (Class III). In comparison with autografts, biosynthetic skin substitutes and human cadaver skin showed comparable efficacy in early reconstructions. Nevertheless, there is currently no skin substitute that would have all the properties of human skin. With the use of substitutes, it is often possible to achieve tissue healing, but many skin functions (sensation, thermoregulation, secretion or UV protection) cannot be restored [59].

### 4.5. Sepsis

Given that sepsis plays a significant role in increasing mortality associated with burns and hypermetabolic response, every effort should be made to control its rate by taking appropriate measures. These will also help prevent infection in patients [60].

Prevention and early recognition of sepsis is a key element in the critical care of the burn patient [60]. Prevention strategies include topical antimicrobial dressings, early excision and grafting, as well as nutritional support. In patients with contaminated wounds or immunocompromised patients, such as diabetic patients, children, and perioperative patients, it is recommended to perform a bacterial culture of the wound and, as an option, introduce antibiotic prophylaxis [50]. However, preventive systemic administration of antibiotics is not (currently) recommended due to the absence of sufficient evidence to support its effectiveness. Nevertheless, burn infections should be proactively treated with a systemic antibiotic therapy and, if necessary, antifungal agents. The treatment is becoming increasingly difficult due to the drug resistance of many bacterial strains. Pro-calcitonin, white blood cell count and C-reactive protein (CRP) levels are, at present, the most commonly used markers of sepsis [61]. In patients with burns, procalcitonin was moderately sensitive (73%) and specific (75%) for sepsis, white blood cell count had poor sensitivity (47%) and moderate specificity (65%), and C-reactive protein was highly sensitive (86%) but poorly specific (54%). Other biomarkers such as stroke volume index, brain natriuretic peptide, TNF-alpha, and cell-free DNA measured on day 14 post injury showed the most promise [60].

### 4.6. Thermoregulation

Another conservative therapeutic measure that helps reduce resting energy expenditure in burn patients with more than 40% TBSA is to increase room temperature. This simple action raises the patient’s core body temperature, thus reducing body water loss. Severe physiological changes following severe thermal injury disrupt thermal homeostasis and render burn patients susceptible to hypothermia. Raising the ambient temperature in the operating room and intensive care unit can mitigate the loss of thermoregulation, prevent hypothermia and minimise the impact of hypermetabolism. However, there is still no conclusive scientific support for this recommendation [62].

### 4.7. Treatment of Contractures

Burn wound contracture is an inevitable consequence of burn injury, which, if not properly treated, remains with the patient for life. It can be prevented with early, progressive physical therapy using specific regimens aimed at improving body mass and muscle strength. There are many therapies designed to reduce contractures, including corticosteroid injection, antihistamine administration, hydrotherapy, dynamic or static splinting, laser therapy, compression therapy, as well as surgical excision and reconstruction [63].

### 4.8. Hormonal Regulation

Attempts to modulate burn-induced hormonal deregulation have led to the establishment of several pharmacological therapeutic strategies. They can be classified according to the use of anabolic agents or anti-catabolic agents. The former include GH, insulin, IGF-1, oxandrolone and testosterone. In turn, propranolol, an adrenergic antagonist, remains the most important anti-catabolic agent [26,64].

Recombinant human growth hormone (rhGH) is an effective modulator of the burn-initiated response with a broad spectrum of action. It suppresses the hepatic acute phase response by increasing the levels of constitutive hepatic proteins, reduces acute phase proteins and modulates cytokine expression, as well as reducing healing time of the skin graft site, improving muscle protein kinetics and maintaining anabolic muscular growth [26]. It also stimulates the production of many other proteins and attenuates nitrogen loss after injury. However, rhGH treatment has been linked to an increased risk of fatal complications in adult patients, which significantly restricts its administration [64].

In turn, recombinant human IGF-1 and IGFBP-3 have been shown to effectively improve muscle protein synthesis in burn patients with significantly fewer adverse effects compared to GH. In addition, these agents improve intestinal mucosal integrity in severe thermal trauma in children, attenuate muscle catabolism [26], and improve the parameters of hepatic acute phase, inflammatory response and immune response. Given that the clinical use of GH is severely limited, it appears that recombinant human insulin-like growth factor-1 (rhIGF-1) may be a better therapeutic agent to effectively attenuate adverse post-burn reactions [64].

The use of insulin may be recommended in some burn injuries. It prevents muscle catabolism by activating anabolic processes in muscle [26] and preserves lean body mass without increasing hepatic triglyceride production. Apart from its ability to lower blood glucose levels by mediating peripheral glucose uptake into skeletal muscle and adipose tissue and suppressing hepatic gluconeogenesis, insulin is known to enhance DNA replication and protein synthesis by controlling amino acid uptake, increasing fatty acid synthesis and decreasing proteolysis. Insulin administration during hospitalisation has been shown to be very helpful in the treatment of hyperglycaemia in severely burned patients, by improving muscle protein synthesis, accelerating wound healing time and mitigating loss of lean body mass and acute phase response [64,65].

In turn, oxandrolone has found moderate clinical use in the prevention and treatment of the effects of burn injuries. As a synthetic analogue of testosterone, it restores serum testosterone levels, resulting in a sharp increase in gene expression of enzymes involved in muscle anabolism, as well as a decrease in protein catabolism. In addition to improving muscle protein synthesis, increasing lean body mass and bone mineral content, oxandrolone effectively counteracts the effects of hypermetabolism [26,64].

Due to the deleterious effects induced by elevated body catecholamine levels, anti-catabolic agents have been introduced into the burn injury management protocol. Propranolol has been shown to reduce compulsory thermogenesis, resting energy expenditure and tachycardia. It has also been found to play a role in increasing lean body mass, decreasing urinary nitrogen loss and urea production. It also reduces insulin resistance, peripheral lipolysis, hepatic acute phase protein response, fatty infiltration of the liver and skeletal muscle wasting [64].

### 4.9. Nutrition in Burn Patients

**Figure 3:**
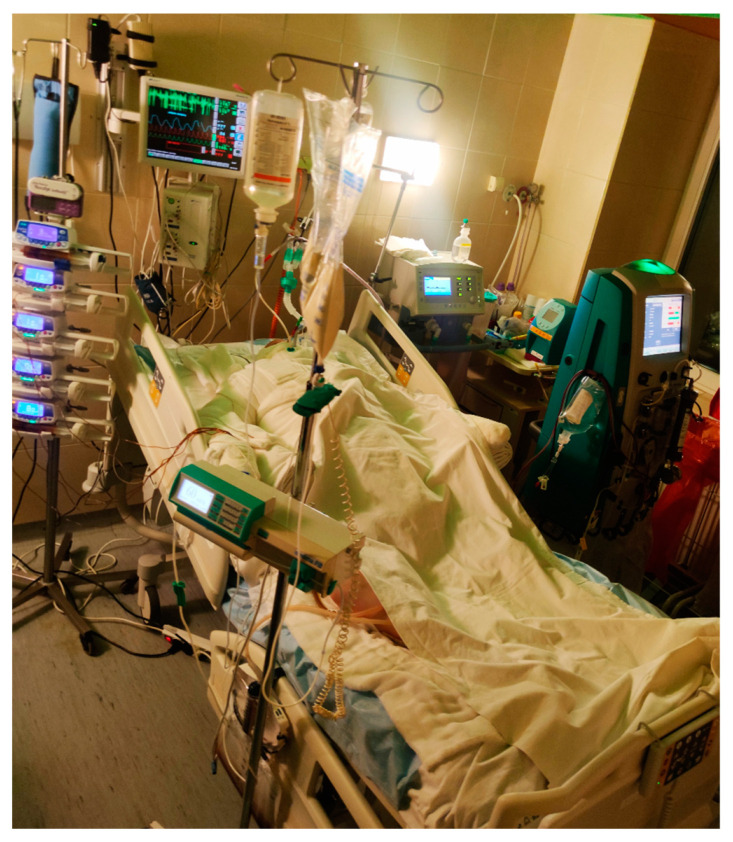
Alternative nutrition in patients with burn injury.

Implementation of well-balanced alternative nutrition is of utmost importance in the recovery process of burn patients (Figure 3). Enteral nutrition has become the gold standard, in contrast to oral nutrition alone, as it usually succeeds in preserving total body weight and attenuates hypermetabolic response in burn patients [66]. It also helps preserve gastrointestinal motility and lowers the risk of sepsis [67]. In the presence of absolute contraindications for enteral feeding, such as prolonged ileus and intolerance to enteral feeding, or in cases where enteral nutrition alone fails to achieve the required calorie intake, parenteral feeding can be considered, but it is not universally recommended due to its possible side effects that include immunosuppression, impaired liver function and increased mortality. When it comes to the dietary profile best suited to the needs of burn patients, there are several factors to consider in order to maintain lean body mass. Given the high rates of amino acid oxidation in burn patients, it may be advisable to stimulate protein synthesis and maintain lean body mass with a high protein, high carbohydrate diet, which also increases endogenous insulin production [67,68].

#### 4.9.1. Duration of Nutritional Support

Time to nutrition is an important factor affecting patient survival after severe burn injuries [69]. Significant damage to the intestinal mucosa and increased bacterial translocation that occur after a burn injury result in decreased nutrient absorption [67,69]. For this reason, nutritional support should preferably be started within 24 h of injury [70]. In animal models, early enteral feeding has been shown to significantly reduce the hypermetabolic response after severe burns [66]. Such a marked improvement in the hypermetabolic response has not been corroborated in human studies; however, early enteral feeding has been linked to a decrease in circulating catecholamines, cortisol and glucagon and preservation of intestinal mucosal integrity [69,71,72]. Early enteral feeding in humans also results in better muscle mass maintenance, accelerated wound healing, lower risk of Curling ulcer formation and shorter intensive care unit stays [68]. Nutrition, both enteral and parenteral, is generally administered in a continuous fashion. In the case of parenteral nutrition, this is carried out for logistical reasons, but with enteral nutrition, the reasons for continuous feeding are less clear. Initially, enteral feeding is administered continuously at a low volume, gradually working towards the target volume to ensure that the patient can tolerate the regimen. The continuous feeding schedule is usually continued even if the patient has no tolerance issues. Continuous enteral feeding is likely to be a remnant of parenteral schedules and to date, the superiority of either schedule has not been supported by data, albeit limited [73].

#### 4.9.2. The Role of Micronutrients in the Nutrition of Burn Patients

Maintaining a healthy immune function and good wound healing is crucially important in patients following burn injury [69]. To this end, it is necessary to maintain normal metabolism of many vitamins and trace elements involved in these processes and to provide for sufficient nutrient intake to meet the increased energy requirements during this time [74]. Major burns trigger severe oxidative stress, which, combined with the substantial inflammatory response, contributes to the depletion of endogenous antioxidants, which in turn are highly dependent on adequate micronutrient concentrations [69,75,76].

In burns greater than 20% TBSA, it is very common to observe significant and fast progressing deficiencies of micro- and macroelements, which are associated with the production of large amounts of burn wound exudate. Significant amounts of Fe, Cu, Se and Zn have been found in exudative fluids [77]. One well-established strategy that has been observed to result in improved wound healing and fewer infectious complications is early intravenous supplementation, recommended by professional organisations in both Europe (European Society for Clinical Nutrition and Metabolism, European Burns Association) and America (American Burns Association). In this context, it is also vitally important to provide for weekly monitoring of serum levels of specific elements in such patients, or at the very least in those with burns exceeding 40% TBSA. It has been shown that in severe burns, such an approach makes it possible to detect pathologically low levels of these elements and intervene in a timely manner with intravenous supplementation [77].

Among the essential minerals is zinc, which plays a key role in wound healing, lymphocyte function, DNA replication and protein synthesis [74,78]. Iron acts as a cofactor for proteins involved in oxygen transport, while selenium improves cell-mediated immunity [75]. Copper is critical for the formation of mature, organised collagen, and its deficiency has been linked to cardiac arrhythmias, impaired immunity, and worse outcomes after burns [79].

Deficient levels of vitamins A, C, and D, as well as Fe, Cu, Se, and Zn, have been shown to adversely affect wound healing rates and skeletal muscle metabolism, as well as impair immune function [80,81]. Vitamin A accelerates wound healing by stimulating epithelial growth, while vitamin C promotes collagen maturation and cross-linking [82]. Vitamin D contributes to bone density and has been observed to be deficient after burns, but its exact role and optimal dose after severe burns remain unclear.

In paediatric burn patients, Ca and vitamin D homeostasis may be significantly dysfunctional for a number of reasons. Children with severe burns have increased bone resorption, osteoblast apoptosis, and urinary excretion of Ca. In addition, burned skin is unable to synthesise normal amounts of vitamin D3, leading to further deregulation in Ca and vitamin D levels. A study of children with burns showed that supplementation with a multivitamin containing 400 IU of vitamin D2 failed to correct vitamin D deficiency [83].

## Data Availability

Not applicable.
